# Utilization of Telemedicine during COVID-19 in Saudi Arabia: A Multicenter Study

**DOI:** 10.7759/cureus.41541

**Published:** 2023-07-07

**Authors:** Abbas Al Mutair, Chandni Saha, Waad Alhuqbani, Mohammed N Alhuqbani, Mohammed N AlQahtani, Ahmad K Abogosh, Abdulaziz M Alsedrah, Alanoud H Alhindi, Reema H Alfehaid, Awad Al-omari

**Affiliations:** 1 Research Center, Almoosa Health Group, Al Ahsa, SAU; 2 Research, King Faisal Specialist Hospital and Research Centre, Riyadh, SAU; 3 Research Center, Dr. Sulaiman Al Habib Medical Group, Riyadh, SAU

**Keywords:** retrospective study, home health care, telemedicine, saudi arabia, covid-19

## Abstract

Background: The outbreak of the novel Coronavirus disease 2019 (COVID-19) has influenced all aspects of life and significantly impacted healthcare services. It has collectively necessitated the use of telemedicine in providing healthcare. Through this study, we aim to report the statistics on telemedicine utilization and satisfaction across the Kingdom of Saudi Arabia during COVID-19.

Methods: This is a cross-sectional study to report the utilization and patient satisfaction with telemedicine services across Saudi Arabia. The data was collected retrospectively from March 2020 to July 2020 on 22,620 patients who used telemedicine services for consultations, medicine refills, and home healthcare visits during COVID-19.

Results: The patients received a quick response to their calls within a mean (± SD) waiting time of 2.54 (± 6.8) minutes corresponding to a median (IQR) of 0 (0-1) minutes. Home healthcare services were presented within a median (IQR) time of 20.16 (4.64 - 42.28) hours, and patients received medication at home with a median (IQR) time of 18.8 (12.15 - 36.1) hours. Conversations over the phone varied for a median (IQR) time of 5 (3-7) minutes. The highest number of telemedicine calls were for family medicine consultations, i.e., 6729 (29.7%), and the lowest was for infectious diseases 04 (0.1%), followed by cardiology consultations, i.e., 635 (2.8%). A total of 13,154 (58.15) rated their overall satisfaction, of which 11,684 (88.82%) found telemedicine services satisfactory.

Conclusion: The utilization of telemedicine across Saudi Arabia results have shown telemedicine to be a satisfactory service for convenient and safe communication between patients and their healthcare providers. It can thus be established as a smart and indissoluble service across the kingdom. However, there is a need to raise awareness of insurance coverage for such services to make them more feasible and accessible to the public.

## Introduction

The coronavirus disease 2019 (COVID-19) pandemic has drained physicians, hospitals, and healthcare institutes. This overwhelming pressure has rapidly shifted healthcare delivery to telemedicine in response to the increased demand for primary care and patients' needs for healthcare services at their convenience. Telemedicine embraces electronic communication technologies to provide healthcare services to patients regardless of geographical barriers [1]. Oxford defines it as "the remote diagnosis and treatment of patients by means of telecommunications technology" [2]. 

However, telemedicine is not a new concept in the medical community; it has only resurfaced during the COVID-19 pandemic. A book on telemedicine sheds light on the fact that clinicians, researchers in health services, and others have been studying how to employ cutting-edge computer and telecommunications technology to enhance healthcare for more than 50 years. Telemedicine is a product of this advanced information technology's intersection and is central to many smart initiatives [3]. As healthcare systems worldwide are undergoing profound changes, telemedicine is gaining attraction beyond its demand in rural areas. The National Center for Health Statistics, USA 2021-22 reports 37% of adults used telemedicine [4]. Before COVID-19, only 11 visits per 1,000 patients in rural and seven visits per 1,000 patients in urban regions were made via telemedicine in Ontario, Canada. Following COVID-19, telemedicine visits rose to 147 per 1,000 patients in rural areas and 220 per 1,000 patients in urban areas. From 2020 to 2025, India projects that the telemedicine market will grow by 31% [5]. Similarly, among the Organization for Economic Cooperation and Development (OECD) countries, 23 out of 31 countries allow teleconsultations by health workers other than doctors, six more than before the COVID‑19 pandemic [6]. A report by McKinsey & Company summarized increased healthcare access, convenience, a platform to apply new medical learning to treat patients across the globe, ability to decrease expenses generated by unnecessary physician visits as some of the significant benefits of telemedicine that facilitated its rapid growth. The report adds, by providing virtual urgent care support, 20% of all trips to emergency rooms can be avoided. It also discovered that 24% of outpatient and office visits in the healthcare industry could be handled remotely with telemedicine. Cost-wise, this amounts to $250 billion in 2020 healthcare expenses that may be virtually transferred. The report also presented some of the cons of telemedicine: inaccessibility in the homes of older, ethnic, and other minority groups or/and who are at the lower end of the socio-economic status due to barriers in obtaining the technology, more training required for an advanced telemedicine platform than for a straightforward mobile health device that is utilized between primary care physicians and specialists and language barriers. A study involving 3,000 cardiology patients at the University of Pennsylvania showed 54% canceled or failed to attend their telemedicine session, reportedly due to patients not speaking English (common language) as their primary language [7]. Further, reduced in-person interaction was one of telemedicine's most frequently reported drawbacks [8]. However, a randomized controlled trial comparing in-person vs. telemedicine for follow-up visits for overactive bladder with 48 patients showed no significant difference between the group in terms of patient satisfaction [9], and in terms of cost-effectiveness, it reduces the overall cost compared to in-person-based care [10].

The growth of telemedicine in Saudi Arabia cannot be denied as well. In Saudi Arabia, Vision 2030 set in motion the workflow digitization of public and private healthcare providers with electronic health records (EHRs) and clinical workflow management systems [11]. This provided the required impetus for telemedicine to thrive. More recently, the pandemic compelled Saudi Arabia to implement digital technologies centered on consumer needs, including telemedicine. The government launched the Sehhaty platform and the 937 Call Center to facilitate telemedicine across the kingdom, among other e-health services. Despite these efforts, the reports on the utilization of telemedicine across the kingdom are limited to a few sporadic studies, even among which the reported results are either from a small sample size or ambiguous. In a study on telemedicine use in Saudi Arabia, of 781 participants from both urban and rural areas, 70% acknowledged the benefits of telemedicine; however, 51% never used phones to seek medical advice. Thus, through this multicenter study, we aim to present a vivid picture of the extent of utilization of telemedicine and patient satisfaction with service across Saudi Arabia.

## Materials and methods

Study design, setting, and participants

This multicenter cross-sectional study looks at retrospective data from six tertiary care hospitals across Saudi Arabia. Data were extracted from the electronic call records of patients who accessed telemedicine services from March 2020 to July 2020 to analyze the utilization and patient satisfaction with telemedicine services during the COVID-19 outbreak. Data extraction included socio-demographic data and data on telemedicine consultations, medicine refills, home health services, and patients' ratings at the end of these calls on their satisfaction level. The study included data from all patients who used only telemedicine services during these five months while excluding records of other e-health services (like electronic health records).

Statistical analysis

All collected data were analyzed using the Statistical Package for Social Sciences (SPSS, version 25; IBM Corp., Armonk, NY, USA). Before the statistical analysis, the extracted data was cleaned and coded using Microsoft Excel (Microsoft, Redmond, WA, USA) for completeness. The data was then analyzed descriptively, reporting actual frequencies and percentages. Further, the mean with standard deviation and median with interquartile range (IQR) were also calculated. For inferential statistical analysis, chi-square, t-test, and analysis of variance (ANOVA) were suitably applied to estimate the significance of the data. 

Ethical considerations

As one of the largest retrospective data collections on telemedicine in Saudi Arabia, this study commenced after the IRB approval was obtained in March 2020. As the data collected in this study was from an electronic data recording system, retrospectively, patients were deidentified, and no personal information was extracted. No data was used other than the purpose stated.

## Results

A total of 22,620 patients' records, who received consultation through telemedicine services were analyzed in this study, of which 13,390 (59.2%) patients were females, 19,290 (85.3%) patients were Saudi citizens, 7,180 (34.5%) were married, 6,729 (29.7%) called for family medicine while only four (0.1%) called infectious disease clinics and 635 (2.8%) for cardiology. Further, among these 22,620 patients, 6,102 (27%) requested medication and 434 (1.9%) home healthcare. 2,605 (11.5%) were asked for blood tests, 699 (3.1%) for radiology tests, and 15,204 (67.2%) were prescribed medications. Overall satisfaction rating was provided by 13,154 (58.15%) of the patients; among them, 11,684 (88.82%) gave a satisfactory rating. However, only marital status, type of clinic, nationality, and medication prescription showed significant differences in patient ratings (Table 1). The Tukey post hoc test revealed that married and single had significantly lower ratings [mean (SD) 2.54 (± 2.32) and 2.72 (± 2.28) respectively] when compared to divorced and widowed [mean (SD) 2.74 (± 2.30) and 3.38 (± 2.03) respectively]. Despite the statistical significance, it cannot be considered clinically significant as a large proportion of marital status was not recorded [10,383 (45.9)]. With the type of clinic, the Tukey post hoc test revealed that family medicine, pediatric, dermatology, and OBGYN telemedicine services had significantly higher ratings. Similarly, for nationality, Saudis rated significantly higher than non-Saudis.

**Table 1:** Basic characteristics measured as categorical variables (n = 22,620)

Characteristics	N (%)	P value
Gender		0.162
Males	9230 (40.8%)	
Females	13390 (59.2%)	
Nationality		0.038
Data Not Available	14 (0.1%)	
Saudi	19290 (85.3%)	
Non-Saudi	3316 (14.6%)	
Marital status		>0.0001
Married	7180 (31.7%)	
Single	4767 (21.1%)	
Divorced	244 (1.1%)	
Widowed	46 (0.2%)	
No response	10383 (45.9%)	
Patient called for medication		0.086
Yes	6102 (27%)	
No	16518 (73%)	
Patient has home health care request		0.001
Yes	434 (1.9%)	
No	22186 (98.1%)	
Type of clinic (specialty)		>0.0001
Family Medicine	6729 (29.7%)	
OBGYNs	4069 (18.0%)	
Pediatric	3643 (16.1%)	
Dermatology	3268 (14.4%)	
Endocrinology	1511 (6.7%)	
ENT	1961 (8.7%)	
Ophthalmology	800 (3.5%)	
Cardiology	635 (2.8%)	
Infectious Diseases	04 (0.1%)	
Blood test ordered		0.066
Yes	2605 (11.5%)	
No	20015 (88.5 %)	
Radiology test ordered		0.514
Yes	699 (3.1%)	
No	21921 (96.6%)	
Medication prescribed		>0.0001
Yes	15204 (67.2%)	
No	7416 (32.8%)	
Type of payment		
Cash	22620 (100.0%)	
Insurance and others	0	
Rating for the received health care services		
1	216 (1.0%)	
2	338 (1.5%)	
3	916 (4.0%)	
4	3201 (14.2%)	
5	8483 (37.5%)	
No rating	9466 (41.8%)	

The median (IQR) age of patients who used telemedicine services was 32 (24-40) years. These patients received a quick response to their calls with a median (IQR) waiting time of 0 (0-1) minutes; however, the mean waiting time was reported to be 2.54 (± 6.8) minutes. Home healthcare services attended to the need within a median (IQR) time of 20.16 (4.64 - 42.28) hours, and patients received medication at home within a median (IQR) time of 18.8 (12.15 - 36.1) hours. Conversations over the phone varied and lasted for a median time of 5 (3-7) minutes. It was analyzed from inferential statistics that none of these, age, waiting time, etc., showed any significant difference in satisfaction rating except for waiting time (Table 2). For waiting time, the correlation (R) revealed an inverse relation between waiting time and satisfaction rating; however, the correlation was not strong (R = -0.042).

**Table 2:** Basic characteristics measured as continuous variables (n=22,620) IQR: interquartile range

Characteristics	Median (IQR)	P
Age	32 (24-40) years	0.195
Waiting time for patients calling for services	0 (0-1) minutes	> 0.0001
Call duration (conversation with patient)	5 (3-7) minutes	0.214
Waiting time for patient to receive home health care	20.16 (4.64 – 42.28) hours	0.459
Waiting time for patient to receive medications at home	18.8 (12.15 – 36.1) hours	0.156

## Discussion

Nowadays, digital technology can equalize the relationship between healthcare providers and patients to act faster and more effectively in consultations about diseases. Telemedicine has been well-perceived by both physicians and patients across Saudi Arabia. Kaliyadan et al. (2020) reported that 58.1% of physicians used telemedicine [12]. In this study, 22,620 patients accessed telemedicine services in just five months in 2020 during COVID-19, among whom 18,438 (79.3%) of the patients found telemedicine services satisfactory. Such high utilization and rating are probably due to the government's efforts to facilitate and raise awareness of telehealth services across the kingdom [13]. The understanding and utilization of telemedicine in Saudi Arabia proliferated during COVID-19 compared to pre-COVID-19, from 46% to 78% (awareness) and 2% to 48% (utilization) [14]. The global telemedicine market just for teleconsultation was valued at USD 29.5 billion in 2022 and is expected to rise further [15]. Additionally, though a report suggested growing geriatric population and the need for home-based services were some of the key contributors to the growth in this sector [15], in our study, the utilization of home-health services was requested by just 434 (1.9%), and the average (median) age of patients using the telemedicine service was 32 years. This, however, is corroborated by another study from Saudi Arabia conducted by Al-Rayes et al., 2021. This study on public awareness of telemedicine in Saudi Arabia reported the most aware age group was between 20 and 39 [14].

Further, telemedicine came in handy when the world grappled with several COVID-19 restrictions. From hand hygiene to social distancing and quarantine [16], COVID-19 preventive measures rapidly escalated. These restrictions impacted the delivery of healthcare services worldwide. For instance, compared to the same month in 2019, the quantity of in-person primary care consultations fell precipitously in May 2020. It decreased by 66% in Portugal, roughly 40% in Australia, 18% in Austria, and 7% in Norway [17]. According to the Centers for Disease Control and Prevention (CDC) Morbidity Mortality Weekly Reports, almost 40% of individuals in the US delayed seeking medical attention due to COVID-19 [18]. However, early in the pandemic, teleconsultations proliferated, somewhat offsetting the decline in in-person healthcare services [18]. The CDC National Health Interview Survey, as cited earlier, shows in 2021, 37.0% of adults in the United States of America (USA) used telemedicine in the past 12 months. It provided continuous health care to patients without their presence in healthcare facilities when local hospitals and healthcare centers did not meet their needs due to COVID-19 cases [19,20]. Therefore, it reduced the risk of transmission because patients had limited exposure to infections [19]. In Saudi Arabia with the MOH launching telehealth services facilitated the rapid increase of telehealth users and presently has more than 2 million users [21]. According to a study, 346 of 402 (86%) respondents admitted that these services could be helpful and had already used them to schedule appointments for their kids [22]. Another study with 781 participants found that 392 of 781 (51%) of the users were satisfied with the telehealth services provided by MOH [23]. In our study, 13,154 (58.15%) rated their experience; among them, most of them, 11,684 (88.82%), found telemedicine services satisfactory. Of the 11,684 patients, more than 60% found it highly satisfactory and rated 5 on 5, while only 1% (225) of the total users (22,620) ranked their satisfaction as low as 1 and 9,466 (41.85%) did not rank at all. These findings correspond to similar results in a study measuring patients' satisfaction with telemedicine in the COVID-19 in Saudi Arabia [19]. However, only nationality, medication prescribed, type of clinic, and marital status led to a significant impact on satisfaction rating. As most of our participants were Saudi nationals, ease of language and ability to communicate in the local language could have impacted their rating; as mentioned earlier, being unable to share in the local language is a barrier to telecommunication [7]. In a similar data analysis scale, a US study analyzed 28,222 telemedicine encounters and reported high satisfaction positively correlated with prescription receipt [24]. According to the study, telemedicine is a substitute for physician visits, and if medications can be prescribed through telemedicine, it leads to better satisfaction. This could be true in COVID-19 time, as people were either restricted by local laws or preferred not to go out due to the circumstances. Regarding the type of clinic, in our study, the most frequently requested clinics were family medicine at 6,729 (29.7%). Telemedicine is known to be successful in managing chronic conditions and common infections; where patients can contact their healthcare providers with the use of live video or a phone call which can be very useful for healthcare professionals to assess their patients by acquiring the essential questions and provide a proper consultation as several studies have reported [25-27]. This was followed by obstetrics and gynecology (OBGYN) (4,069, 18.0%), and Greiner 2017 [28] explains telehealth in OBGNY is helpful in prenatal treatment, mental health care, genetic counseling, fetal echocardiography, monitoring of chronic medical problems in pregnancy, colposcopy, and medical abortion and reported that underserved women who used telemedicine primarily for outpatient gynecologic visits had positive experiences during COVID-19 [28]. Our results on marital status significantly impacting rating lack clinical significance as in most patients (10,383; 45.9%), marital status was not recorded. Though not significantly impacting rating, our analysis also reports 13,390 (59.2%) of the users were females, and per the CDC, in the US, age-related increases in telemedicine use were seen, and women (42.0%) used it more frequently than males (31.7%) in 2021.

In an exclusive finding, we observed that all of the patients used cash as a mode of payment for telemedicine services, and none of them used insurance. However, later that year, in June 2020, the Council of Cooperative Health Insurance (CCHI) Saudi Arabia, in their efforts to make telemedicine feasible to all, issued the approval of the telemedicine service coverage within the benefits of the standard health insurance policy in 2020 with the rise of COVID-19 [29]. Even in the US, insurance services have just started to cover telemedicine in 2020 [30]. Patients must be informed of such facilities by their insurance companies and healthcare providers to use such novel services best. The previously cited study on 28,222 telemedicine encounters also states that telemedicine insurance coverage has not kept pace with its expansion. Finally, this study also highlights the swiftness of the telemedicine service in Saudi Arabia, with almost zero waiting time which also was inversely correlated with the service rating; however, this correlation was not strong enough to conclude that lower waiting time corresponds to high satisfaction.

As a limitation, this study cannot be generalized to all clinics as it only included family medicine, OBGYN, pediatrics, dermatology, endocrinology, ENT, ophthalmology, cardiology, and a tiny fraction of infectious diseases. Nevertheless, observing the support from the government, growing demand for telemedicine services, and satisfaction with telemedicine in Saudi Arabia, it is safe to recommend the implementation of telemedicine as a routine service across the kingdom.

## Conclusions

The COVID-19 pandemic has propelled digital technology to deliver healthcare services. As innovation in healthcare services increases, it becomes necessary to evaluate and understand the existing utilization and satisfaction related to such services to estimate its expansion in the future. This multicenter study presents a comprehensive status of telemedicine utilization and satisfaction across the Kingdom of Saudi Arabia. The results of this study could be valuable to the healthcare sector looking forward to implementing and investing in routine e-healthcare services. The utilization of telemedicine across Saudi Arabia results have shown telemedicine to be a practical approach for convenient and safe communication between patients and their healthcare providers. However, to increase the feasibility, insurance companies and healthcare providers must raise awareness of insurance coverage of such services.
